# Integrated First-Trimester Assessment of Placental Biomarkers, Metabolic Indices, and IGF-1/IGFBP-5 in Relation to Subsequent Gestational Diabetes Mellitus: A Prospective Cohort Study

**DOI:** 10.3390/medicina62091804

**Published:** 2026-09-19

**Authors:** Soner Gök, Berfin Can Gök, Esin Avcı Çiçek, Hande Senol

**Affiliations:** 1 Department of Obstetrics and Gynecology, School of Medicine, Pamukkale University, 20160 Denizli, Turkey; 2 Department of Obstetrics and Gynecology, Denizli State Hospital, 20010 Denizli, Turkey; berfincan.gok@saglik.gov.tr; 3 Department of Biochemistry, School of Medicine, Pamukkale University, 20160 Denizli, Turkey; eavci@pau.edu.tr; 4 Department of Biostatistics, School of Medicine, Pamukkale University, 20160 Denizli, Turkey; hsenol@pau.edu.tr

**Keywords:** gestational diabetes mellitus, first-trimester screening, PAPP-A, HOMA-IR, TyG index, IGF-1, IGFBP-5, risk assessment

## Abstract

*Background and Objectives*: Gestational diabetes mellitus (GDM) is usually diagnosed at 24–28 gestational weeks, although metabolic and placental alterations may emerge considerably earlier. This study evaluated whether routinely available first-trimester placental biomarkers, metabolic indices, and insulin-like growth factor (IGF)-axis components are associated with GDM diagnosed later in pregnancy and whether these markers provide complementary information across distinct biological domains. *Materials and Methods*: Of 200 women completing the 75-g OGTT (34 with GDM and 166 normoglycemic), deferred IGF-1 and IGFBP-5 assays were performed in a complete-biomarker analytic cohort of 90 participants with complete clinical follow-up and available stored specimens (30 GDM and 60 controls). At 11+0–13+6 weeks, pregnancy-associated plasma protein-A (PAPP-A), free β-human chorionic gonadotropin (free β-hCG), IGF-1, IGF-binding protein-5 (IGFBP-5), fasting glucose, insulin, and lipid parameters were assessed. HOMA-IR, the triglyceride–glucose (TyG) index, TG/HDL-C, and LDL/HDL-C were calculated. GDM was diagnosed using a 75-g oral glucose tolerance test at 24–28 weeks. Group comparisons, correlation analyses, and logistic regression were performed. *Results*: Compared with controls, women who later developed GDM had higher first-trimester insulin, HOMA-IR, triglycerides, VLDL-C, TyG, and TG/HDL-C and lower HDL-C, free β-hCG, PAPP-A, PAPP-A MoM, and IGF-1 (all *p* < 0.05). Birth weight was also higher in the GDM group. Correlation patterns differed between groups, suggesting distinct relationships among placental, metabolic, and IGF-related markers. In the four-variable adjusted model, higher HOMA-IR (OR 2.123, 95% CI 1.089–4.137; *p* = 0.027) and TyG (OR 3.747, 95% CI 1.010–13.894; *p* = 0.048) were associated with increased GDM odds, whereas free β-hCG MoM (OR 0.325, 95% CI 0.115–0.920; *p* = 0.034) and IGF-1 (OR 0.856, 95% CI 0.748–0.980; *p* = 0.024) showed inverse associations. *Conclusions*: Pregnancies that subsequently developed GDM showed detectable first-trimester differences across metabolic, placental, and IGF-related pathways. The combined pattern supports further evaluation of multidomain early-risk assessment, although clinical predictive utility requires validation in larger independent cohorts.

## 1. Introduction

Gestational diabetes mellitus (GDM) has implications beyond the period of pregnancy, affecting both immediate obstetric outcomes and later metabolic health. Its reported frequency differs across populations and diagnostic approaches; when International Association of Diabetes and Pregnancy Study Groups (IADPSG) criteria are applied, the recent global estimate is approximately 14% [1]. GDM is associated with hypertensive disorders, operative birth, excessive fetal growth, shoulder dystocia, and neonatal hypoglycemia [2,3]. Mothers also face increased risks of GDM in future pregnancies and type 2 diabetes, while exposed offspring have a higher propensity for obesity and impaired glucose regulation [3,4]. These consequences provide a rationale for examining biological changes before conventional diagnostic testing.

Routine GDM assessment is commonly performed with an oral glucose tolerance test (OGTT) at 24–28 gestational weeks [5,6]. The physiological disturbances that culminate in an abnormal OGTT are likely to develop earlier. Alterations in insulin sensitivity, placental function, inflammatory pathways, and maternal metabolic adaptation can already be present in the first trimester [7,8]. Because this period routinely includes biochemical and ultrasonographic screening, it provides a useful setting in which to investigate markers that precede overt hyperglycemia [9,10].

Among routinely measured first-trimester placental markers, PAPP-A and free β-hCG have attracted attention as possible indicators of later GDM. Both arise from trophoblastic or placental processes, making early differences biologically plausible before glucose intolerance becomes clinically evident [10,11,12,13]. The literature has shown a comparatively consistent tendency toward lower PAPP-A in pregnancies subsequently complicated by GDM, whereas results for free β-hCG are heterogeneous [13,14,15]. Variation in study populations, diagnostic definitions, assays, and baseline risk may contribute to these discrepancies. Available evidence also indicates that neither marker is sufficiently informative as a stand-alone measure.

Maternal lipid metabolism offers another perspective on early metabolic susceptibility. Normal pregnancy requires substantial adaptation of triglyceride and lipoprotein handling, but an exaggerated response early in gestation has been linked to insulin resistance, oxidative and vascular stress, and chronic low-grade inflammation [7,8,16,17]. Consequently, indices that combine glucose and lipid measurements may summarize metabolic disturbance more effectively than isolated analytes.

The triglyceride–glucose (TyG) index is one such surrogate of insulin-resistant metabolism, incorporating fasting glucose and triglyceride concentrations. TG/HDL-C and LDL/HDL-C provide complementary descriptions of the lipid profile and have likewise been evaluated in relation to GDM [18,19,20,21]. These derived measures may reveal an adverse metabolic pattern that is less apparent when individual lipid values are considered separately. However, prospective studies assessing them alongside placental screening biomarkers in early pregnancy remain relatively scarce.

The PAPP-A–IGF axis provides a biological link between placental development and maternal metabolism. By cleaving IGF-binding proteins, particularly IGFBP-4, PAPP-A can increase local availability of IGFs [11,12]. IGF-1 participates in maternal metabolic regulation as well as placental growth, nutrient transport, and fetal development [22], while IGFBP-5 modulates IGF activity and is involved in cellular growth, differentiation, and tissue remodeling [22]. Disturbance of this system could therefore reflect both placental and metabolic abnormalities [11,12,22]. Nevertheless, few prospective pregnancy cohorts have examined placental proteins, routine metabolic indices, and IGF-related biomarkers concurrently [9,14,19,20,22].

We therefore evaluated first-trimester placental screening markers, metabolic indices, IGF-1, and IGFBP-5 in relation to GDM diagnosed later in gestation. Correlations between these variables were also explored, followed by multivariable analysis to determine which associations remained after adjustment. We hypothesized that the different marker groups would reflect partially distinct aspects of early GDM susceptibility and could therefore provide complementary information.

## 2. Materials and Methods

### 2.1. Study Design, Setting, and Ethical Approval

This prospective observational study was conducted at a single tertiary-care center, the Department of Obstetrics and Gynecology, Pamukkale University Hospital, Türkiye. Consecutive women attending routine first-trimester combined aneuploidy screening between September 2021 and August 2023 were invited to participate. Participants were followed through the 24–28-week GDM assessment and subsequently to delivery. The study was reported with reference to STROBE recommendations [23].

Approval was granted by the Pamukkale University Non-Interventional Clinical Research Ethics Committee (Meeting No. 16, 31 August 2021; Document No. E-60116787-020-95762). All procedures conformed to the Declaration of Helsinki and subsequent amendments [24], and written informed consent was obtained before study entry. Consecutive enrollment was used to minimize investigator-driven selection.

### 2.2. Study Population

Potential participants were screened at the 11+0–13+6-week combined aneuploidy visit. Inclusion required age 18–40 years, a viable singleton gestation, completion of the biochemical and ultrasonographic components of first-trimester screening, and written consent. Gestational age based on the last menstrual period was verified using first-trimester crown-rump length (CRL) [25,26]. Enrolled women were then followed until GDM testing and delivery.

Exclusion criteria comprised known type 1 or type 2 diabetes, pre-pregnancy impaired fasting glucose or impaired glucose tolerance [27], a history of gestational diabetes mellitus in a previous pregnancy, multiple pregnancy, chronic hypertension, clinically relevant thyroid or other endocrine disorders (including polycystic ovary syndrome and Cushing syndrome), autoimmune disease, renal or hepatic dysfunction, assisted conception, and use of treatments with substantial effects on glucose metabolism, including systemic corticosteroids. Pregnancies with fetal chromosomal or structural abnormalities, incomplete first-trimester clinical/biochemical/ultrasound information, or no available OGTT result were also excluded.

**Figure 1:**
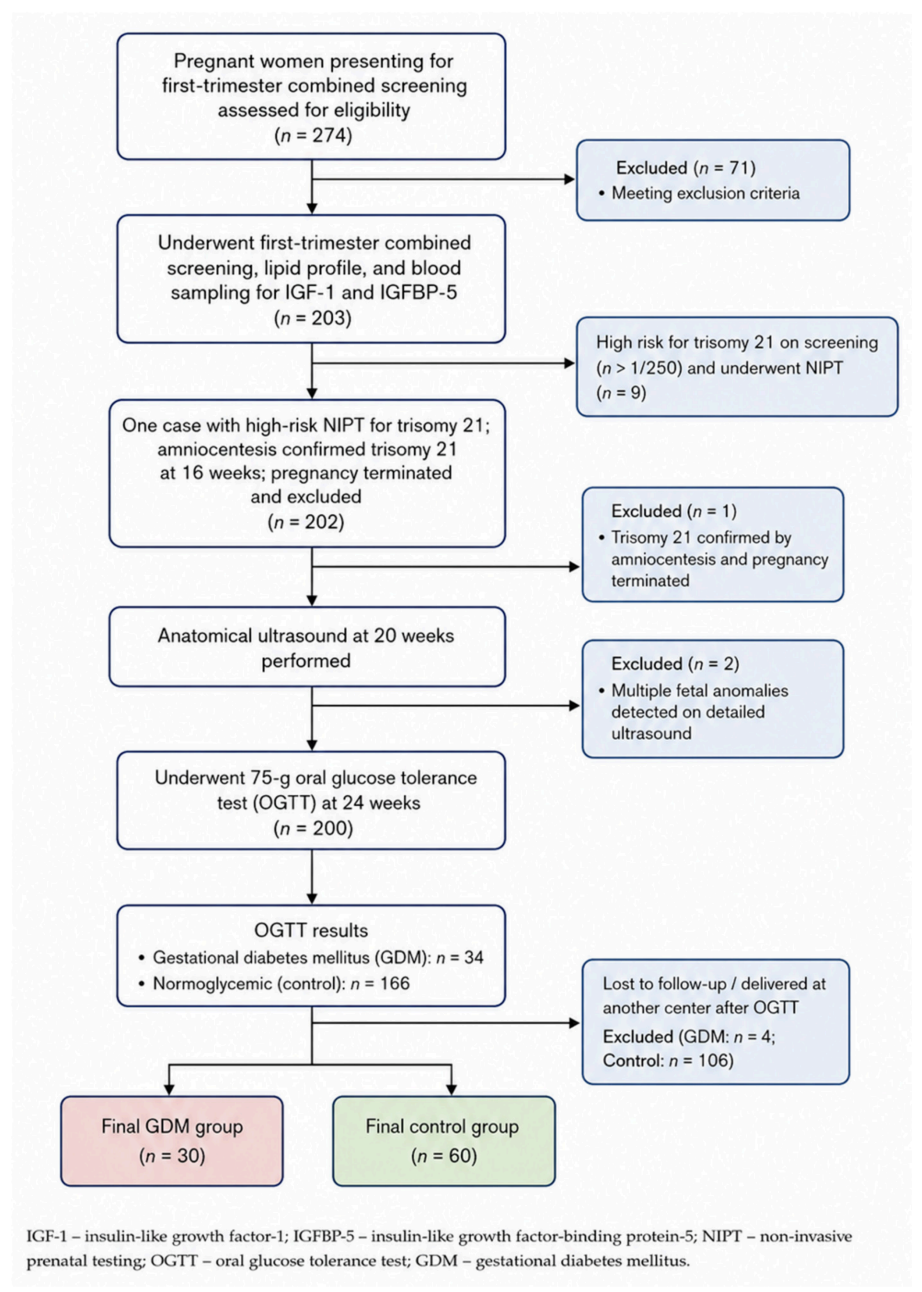
Flowchart of the study population. Abbreviations: GDM, gestational diabetes mellitus; IGF-1, insulin-like growth factor-1; IGFBP-5, insulin-like growth factor-binding protein-5; NIPT, non-invasive prenatal testing; OGTT, oral glucose tolerance test. The flowchart distinguishes the full OGTT cohort (n = 200) from the complete biomarker analytic cohort (n = 90); deferred IGF-1 and IGFBP-5 assays were performed only in the latter group using available stored first-trimester serum specimens.

Of 274 women initially assessed, 71 fulfilled at least one exclusion criterion. The remaining 203 completed first-trimester combined screening and lipid evaluation, and serum intended for subsequent IGF-1 and IGFBP-5 assays was stored at −80 °C. Nine participants with a trisomy 21 screening risk ≥1:250 underwent non-invasive prenatal testing (NIPT). One high-risk NIPT result was confirmed as trisomy 21 by amniocentesis at 16 weeks; following termination at the participant’s request, this pregnancy was excluded from follow-up. Two additional pregnancies were removed after multiple fetal anomalies were detected at the 20-week detailed examination. Thus, 200 women underwent the 75-g OGTT at 24–28 weeks, of whom 34 were diagnosed with GDM and 166 remained normoglycemic. Delivery follow-up was unavailable for four women with GDM and 106 normoglycemic women because they delivered at other institutions. IGF-1 and IGFBP-5 were deferred laboratory measurements performed after completion of clinical follow-up rather than at the time of recruitment. These assays were therefore performed using stored first-trimester serum samples from the 90 participants for whom complete clinical follow-up and stored specimens were available (30 with GDM and 60 normoglycemic controls). IGF-1 and IGFBP-5 were not measured in the remaining 110 women, and their stored serum samples were subsequently discarded; consequently, retrospective completion of the IGF-axis measurements in these participants was not possible. Because the study aimed to evaluate placental, metabolic, and IGF-axis biomarkers concurrently, the primary complete-biomarker analyses were restricted to these 90 participants (Figure 1). Importantly, exclusion of the remaining participants from the complete-biomarker analysis was not based on their OGTT result itself, and GDM status was available for all 200 women who underwent OGTT. Nevertheless, because the proportion retained in the complete-biomarker cohort differed between the GDM and normoglycemic groups, the possibility of differential attrition and selection bias cannot be excluded.

### 2.3. Clinical and Ultrasonographic Assessment

Maternal and obstetric data were prospectively entered on a standardized case-report form at recruitment. Recorded variables included age, gravidity, parity, height, and weight. BMI was obtained by dividing weight in kilograms by height in meters squared [28]. If a reliable pre-pregnancy weight was unavailable, the first-trimester measurement was used.

Combined first-trimester screening was performed between 11+0 and 13+6 weeks, with CRL used to confirm pregnancy dating. All transabdominal scans were performed by one experienced obstetrician under the same institutional protocol to limit measurement variability. NT was assessed in a true midsagittal fetal view with neutral positioning. The mean of three technically acceptable measurements of the maximal translucency between the inner nuchal margins was used in the analysis. Acquisition followed Fetal Medicine Foundation and international recommendations [29,30].

### 2.4. Blood Sampling and Biochemical Analyses

At the 11+0–13+6-week study visit, venous samples were obtained after an overnight fast of 8–12 h and before initiation of any treatment. Collections were scheduled between 08:00 and 10:00 a.m. to limit time-of-day variation in metabolic measurements.

Blood was collected into the appropriate serum or ethylenediaminetetraacetic acid (EDTA) tubes and processed within 60 min. Following centrifugation at 3000 rpm for 10 min, aliquots for routine analyses were tested without delay, while samples reserved for later assays were stored at −80 °C. Additional freeze–thaw cycles were avoided [31].

VLDL-C was directly measured in the Clinical Biochemistry Laboratory.

Fasting glucose, insulin, triglycerides, total cholesterol, HDL-C, and LDL-C were analyzed in the Clinical Biochemistry Laboratory of Pamukkale University Hospital using the laboratory’s routine automated platforms, manufacturer-specified procedures, and internal quality-control practices.

PAPP-A and free β-hCG were quantified in serum by electrochemiluminescence immunoassay on Roche Cobas systems (Mannheim, Germany). Results were expressed as mIU/L for PAPP-A and IU/L for free β-hCG. MoM values were produced with SsdwLab.Ink software 5.0 after adjustment for relevant maternal and pregnancy characteristics, including weight, smoking, ethnicity, diabetes status, and plurality [32].

### 2.5. Measurement of IGF-1 and IGFBP-5

IGF-1 and IGFBP-5 assays were performed in the Research Laboratory, Department of Medical Biochemistry, Pamukkale University Faculty of Medicine. Commercial BT-Lab sandwich ELISA kits (Bioassay Technology Laboratory, Shanghai, China) were applied according to the manufacturer’s protocol [33,34]. Frozen sera were thawed once and allowed to reach room temperature on the day of analysis. Standards and samples were added to the plates and subjected to the specified incubation and washing steps. Absorbance was measured at 450 nm, and concentrations were calculated from the relevant standard curves.

A minimum serum volume of 10 μL was required for each assay. For IGF-1, the manufacturer reported an analytical range of 0.1–40 ng/mL and a detection limit of 0.058 ng/mL; intra-assay and inter-assay coefficients of variation were <10% and <12%, respectively. Serum IGFBP-5 concentrations were measured using a commercial enzyme-linked immunosorbent assay (ELISA) kit (Bioassay Technology Laboratory [BT-Lab], Shanghai, China) with an analytical measurement range of 0.05–20 ng/mL. Prior to testing, serum samples were thawed, mixed thoroughly, and pre-diluted at a 1:5 ratio using the manufacturer’s assay diluent to ensure that the measured concentrations fell within the validated analytical range of the assay. All calibrators and diluted serum samples were analyzed in duplicate by laboratory technicians who were blinded to patient identities and clinical groupings. Absorbances were measured at 450 nm on an automated microplate reader (ELx800, BioTek Instruments Inc., Winooski, VT, USA), and final concentrations were determined by multiplying the interpolated values from a 4-parameter logistic (4-PL) standard curve by the dilution factor (×5). Intra-assay and inter-assay coefficients of variation (CV) were below 8% and 10%, respectively. Concentrations used in the statistical analyses were expressed in ng/mL. Samples were processed using de-identified alphanumeric codes. Duplicate pairs with an analytical CV exceeding 10% were re-tested to ensure measurement precision.

### 2.6. Calculated Metabolic Indices

Fasting biochemical measurements were used to derive indices reflecting insulin resistance and lipid-related metabolic status. TyG was calculated as follows:

TyG = ln [fasting triglycerides (mg/dL) × fasting plasma glucose (mg/dL)/2] [18,35].

TG/HDL-C and LDL/HDL-C were obtained by dividing triglycerides and LDL-C, respectively, by HDL-C. HOMA-IR was calculated from fasting insulin and glucose as [fasting insulin (μU/mL) × fasting plasma glucose (mg/dL)]/405 [36].

### 2.7. Diagnosis and Management of GDM

All participants received a 75-g OGTT between 24 and 28 gestational weeks. According to IADPSG criteria, GDM was diagnosed if at least one glucose value met or exceeded the following threshold: fasting 92 mg/dL (5.1 mmol/L), 1 h 180 mg/dL (10.0 mmol/L), or 2 h 153 mg/dL (8.5 mmol/L) [5,6].

Participants diagnosed with GDM were managed through routine antenatal care, including individualized nutritional counseling, lifestyle intervention, and self-monitoring of blood glucose. Insulin therapy was introduced when glycemic targets were not achieved with medical nutrition therapy [37].

### 2.8. Statistical Analysis

All statistical procedures were performed in IBM SPSS Statistics for Windows, version 25.0 (IBM Corp., Armonk, NY, USA). Continuous-variable distributions were assessed with the Shapiro–Wilk test. Variables approximating a normal distribution are presented as mean ± SD and were compared using the independent-samples Student’s *t*-test; non-normally distributed variables are summarized as median (IQR) and were compared with the Mann–Whitney U test. Categorical data are reported as n (%) and were analyzed with the chi-square test or Fisher’s exact test, as appropriate.

Pearson or Spearman correlation analyses were used, as appropriate, according to data distribution. Binary logistic regression was first performed separately for each first-trimester variable, with subsequent GDM status as the dependent outcome. The primary multivariable model was not strictly prespecified before examination of the data. Candidate variables showing univariable associations at *p* < 0.05 were considered for multivariable analysis together with biological relevance, redundancy, collinearity, and mathematical overlap. Given the limited number of GDM events (n = 30), a parsimonious four-variable model was constructed comprising HOMA-IR, TyG, free β-hCG MoM, and IGF-1, representing complementary metabolic, placental, and IGF-related domains. To reduce redundant information and model complexity, insulin was not entered together with HOMA-IR, triglycerides and TG/HDL-C were not entered with TyG, and absolute free β-hCG was not entered with its MoM value. Regression outputs include β, SE, Wald statistics, OR, 95% CI, and *p* value. Statistical tests were two-sided, with *p* < 0.05 defining significance. Maternal age, BMI, and parity were evaluated in separate sensitivity analyses to assess potential confounding. Variable selection was therefore not based solely on the magnitude of univariable associations. All univariable and multivariable logistic regression analyses were conducted in the complete-biomarker analytic cohort of 90 participants (30 with GDM and 60 normoglycemic controls), with subsequent GDM status entered as the binary dependent outcome; these analyses were not restricted to the GDM subgroup.

Correlation analyses were conducted separately within the GDM and control groups as exploratory descriptive analyses to characterize patterns of within-group associations among placental, metabolic, and IGF-related markers according to subsequent GDM status. These subgroup analyses were not intended as formal tests of between-group differences in correlation coefficients.

Because the limited number of outcome events could increase the risk of small-sample bias, overfitting, and coefficient instability, the stability of the primary four-variable model was additionally evaluated using Firth penalized logistic regression. The same four covariates were entered into the penalized model to permit direct comparison with the conventional logistic regression estimates. Internal coefficient stability was further assessed using 2000 stratified bootstrap resamples, preserving the GDM/control sampling structure. The proportion of bootstrap models retaining the direction of each coefficient observed in the original model was calculated. These analyses were considered sensitivity and internal stability analyses rather than development or validation of a clinical prediction model.

Because HOMA-IR and TyG represent related metabolic constructs and both incorporate fasting glucose, their association was specifically evaluated using Pearson and Spearman correlation analyses. Multicollinearity among the four variables included in the primary multivariable model was further assessed using variance inflation factors (VIFs) and tolerance values.

To assess the robustness of the primary multivariable findings to potential confounding by established maternal GDM risk factors, two additional sensitivity analyses were performed. In Sensitivity Model 1, maternal age and BMI were added simultaneously to the primary four-variable model comprising HOMA-IR, TyG, free β-hCG MoM, and IGF-1. In Sensitivity Model 2, parity was additionally included together with maternal age and BMI. Previous GDM was not evaluated as a covariate because a history of GDM in a previous pregnancy was an exclusion criterion. Family history of diabetes was not systematically recorded and therefore could not be included in the adjusted analyses. Given the limited number of GDM events (n = 30), these expanded models were considered sensitivity analyses rather than primary models. 

## 3. Results

### 3.1. Baseline Clinical, Biochemical, and Pregnancy Characteristics

The analysis included 90 pregnancies, comprising 30 women who subsequently developed GDM and 60 normoglycemic controls (Table 1). During the first trimester, the GDM group had higher insulin, HOMA-IR, triglycerides, VLDL-C, TyG, and TG/HDL-C, while HDL-C, free β-hCG, PAPP-A, PAPP-A MoM, and IGF-1 were lower (all *p* < 0.05). Birth weight was also higher among GDM pregnancies (*p* = 0.012). No statistically significant between-group difference was observed for age, gravidity, parity, BMI, fasting glucose, LDL-C, total cholesterol, LDL/HDL-C, gestational age at screening, free β-hCG MoM, IGFBP-5, or gestational age at delivery. Among the 30 women with GDM in the complete-biomarker analytic cohort, 7 (23.3%) required insulin therapy during pregnancy for glycemic control.

### 3.2. Correlation Analyses

Supplementary Table S1 presents the detailed correlation findings. In women who later developed GDM, free β-hCG correlated positively with PAPP-A MoM (r = 0.452, *p* = 0.012). IGF-1 correlated directly with HDL-C (r = 0.450, *p* = 0.013) and inversely with TG/HDL-C (r = −0.459, *p* = 0.011). More significant relationships were observed in controls, with the largest coefficient between PAPP-A and IGF-1 (r = 0.791, *p* < 0.001). Gestational age at screening was also related to both PAPP-A and PAPP-A MoM in the control group. These group-stratified findings were considered exploratory; no formal inference was made regarding between-group differences in the correlation coefficients.

### 3.3. Logistic Regression Analyses

Logistic regression analyses were performed in the complete-biomarker analytic cohort (N = 90; 30 GDM and 60 controls), rather than within the GDM group alone. The logistic regression results are shown in Table 2. In univariable analyses, higher insulin, HOMA-IR, triglycerides, TyG, and TG/HDL-C corresponded to increased GDM odds, whereas higher HDL-C, free β-hCG, free β-hCG MoM, PAPP-A, PAPP-A MoM, IGF-1, and IGFBP-5 corresponded to lower odds (all *p* < 0.05). In the four-variable adjusted model, HOMA-IR (OR = 2.123, 95% CI: 1.089–4.137; *p* = 0.027) and TyG (OR = 3.747, 95% CI: 1.010–13.894; *p* = 0.048) remained positively associated with GDM. In contrast, free β-hCG MoM (OR = 0.325, 95% CI: 0.115–0.920; *p* = 0.034) and IGF-1 (OR = 0.856, 95% CI: 0.748–0.980; *p* = 0.024) retained inverse associations.

**Table 2:** Univariable and multivariable logistic regression analyses and sensitivity models for subsequent gestational diabetes mellitus.

	Univariable Logistic Regression						Multivariable Logistic Regression					
	β	SE	Wald	*p*	OR	95% CI	β	SE	Wald	*p*	OR	95% CI
Age (years)	0.034	0.043	0.632	0.427	1.035	0.951–1.127	—	—	—	—	—	—
BMI (kg/m^2^)	0.054	0.046	1.387	0.239	1.055	0.965–1.154	—	—	—	—	—	—
Gravida	0.279	0.246	1.293	0.256	1.322	0.817–2.139	—	—	—	—	—	—
Parity	−0.026	0.282	0.009	0.925	0.974	0.560–1.693	—	—	—	—	—	—
Fasting glucose (mg/dL)	0.047	0.030	2.422	0.120	1.048	0.988–1.111	—	—	—	—	—	—
Insulin (μU/mL)	0.165	0.060	7.675	0.006	1.179	1.049–1.325	—	—	—	—	—	—
HOMA-IR	0.724	0.272	7.061	0.008	2.062	1.209–3.516	0.753	0.340	4.891	0.027	2.123	1.089–4.137
HDL-C (mg/dL)	−0.031	0.015	4.116	0.042	0.969	0.940–0.999	—	—	—	—	—	—
LDL-C (mg/dL)	0.006	0.009	0.383	0.536	1.006	0.988–1.023	—	—	—	—	—	—
Total cholesterol (mg/dL)	0.004	0.008	0.265	0.607	1.004	0.989–1.020	—	—	—	—	—	—
Triglycerides (mg/dL)	0.011	0.004	6.719	0.010	1.011	1.003–1.020	—	—	—	—	—	—
TyG index	1.734	0.584	8.823	0.003	5.666	1.804–17.794	1.321	0.669	3.902	0.048	3.747	1.010–13.894
TG/HDL-C ratio	0.470	0.177	7.017	0.008	1.600	1.130–2.266	—	—	—	—	—	—
LDL/HDL-C ratio	0.580	0.319	3.300	0.069	1.787	0.955–3.341	—	—	—	—	—	—
free β-hCG (IU/L)	−0.032	0.015	4.595	0.032	0.968	0.940–0.997	—	—	—	—	—	—
free β-hCG MoM	−0.917	0.433	4.476	0.034	0.400	0.171–0.935	−1.123	0.530	4.486	0.034	0.325	0.115–0.920
PAPP-A (mIU/L)	−0.665	0.226	8.642	0.003	0.514	0.330–0.801	—	—	—	—	—	—
PAPP-A MoM	−1.425	0.659	4.670	0.031	0.241	0.066–0.876	—	—	—	—	—	—
IGF-1 (ng/mL)	−0.165	0.062	7.004	0.008	0.848	0.751–0.958	−0.155	0.069	5.096	0.024	0.856	0.748–0.980
IGFBP-5 (ng/mL)	−0.010	0.005	4.078	0.043	0.990	0.981–1.000	—	—	—	—	—	—
**Sensitivity Model 1: Primary model + maternal age + BMI**												
HOMA-IR	—	—	—	—	—	—	0.802	0.356	5.073	0.024	2.229	1.110–4.478
TyG index	—	—	—	—	—	—	1.387	0.713	3.783	0.052	4.004	0.989–16.207
free β-hCG MoM	—	—	—	—	—	—	−1.118	0.529	4.463	0.035	0.327	0.116–0.922
IGF-1 (ng/mL)	—	—	—	—	—	—	−0.159	0.072	4.923	0.027	0.853	0.741–0.982
Maternal age (years)	—	—	—	—	—	—	0.010	0.054	0.032	0.858	1.010	0.909–1.122
BMI (kg/m^2^)	—	—	—	—	—	—	−0.031	0.058	0.291	0.589	0.969	0.864–1.086
**Sensitivity Model 2: Primary model + maternal age + BMI + parity**												
HOMA-IR	—	—	—	—	—	—	0.780	0.356	4.795	0.029	2.181	1.085–4.382
TyG index	—	—	—	—	—	—	1.452	0.727	3.992	0.046	4.273	1.028–17.757
free β-hCG MoM	—	—	—	—	—	—	−1.138	0.539	4.453	0.035	0.321	0.111–0.922
IGF-1 (ng/mL)	—	—	—	—	—	—	−0.156	0.073	4.616	0.032	0.855	0.742–0.986
Maternal age (years)	—	—	—	—	—	—	0.036	0.067	0.279	0.597	1.036	0.908–1.182
BMI (kg/m^2^)	—	—	—	—	—	—	−0.035	0.059	0.362	0.547	0.965	0.860–1.083
Parity	—	—	—	—	—	—	−0.287	0.448	0.412	0.521	0.750	0.312–1.804

Univariable analyses were performed in the complete cohort (N = 90; 30 GDM and 60 controls). The primary four-variable multivariable model included HOMA-IR, TyG index, free β-hCG MoM, and IGF-1 entered simultaneously. Closely related or mathematically derived variables were not entered together to reduce redundancy and limit overfitting. Results are expressed as β coefficients, SEs, Wald statistics, ORs, 95% CIs, and *p* values. A two-sided *p* < 0.05 indicated statistical significance. Abbreviations: BMI, body mass index; CI, confidence interval; GDM, gestational diabetes mellitus; HDL-C, high-density lipoprotein cholesterol; HOMA-IR, Homeostasis Model Assessment of Insulin Resistance; IGF-1, insulin-like growth factor-1; IGFBP-5, insulin-like growth factor-binding protein-5; LDL-C, low-density lipoprotein cholesterol; MoM, multiples of the median; OR, odds ratio; PAPP-A, pregnancy-associated plasma protein-A; SE, standard error; TG/HDL-C, triglyceride/high-density lipoprotein cholesterol; TyG, triglyceride–glucose; free β-hCG, free β-human chorionic gonadotropin; LDL/HDL-C, low-density lipoprotein/high-density lipoprotein cholesterol.

### 3.4. Sensitivity Analyses for Maternal Clinical Risk Factors

Sensitivity analyses incorporating established maternal GDM risk factors yielded broadly consistent results (Table 2). After additional adjustment for maternal age and BMI, HOMA-IR (OR 2.229, 95% CI 1.110–4.478; *p* = 0.024), free β-hCG MoM (OR 0.327, 95% CI 0.116–0.922; *p* = 0.035), and IGF-1 (OR 0.853, 95% CI 0.741–0.982; *p* = 0.027) remained significantly associated with subsequent GDM, whereas the association for TyG was attenuated slightly and became borderline (OR 4.004, 95% CI 0.989–16.207; *p* = 0.052). Maternal age and BMI were not independently associated with GDM in this model. When parity was additionally included, the associations remained significant for HOMA-IR (OR 2.181, 95% CI 1.085–4.382; *p* = 0.029), TyG (OR 4.273, 95% CI 1.028–17.757; *p* = 0.046), free β-hCG MoM (OR 0.321, 95% CI 0.111–0.922; *p* = 0.035), and IGF-1 (OR 0.855, 95% CI 0.742–0.986; *p* = 0.032), whereas maternal age, BMI, and parity were not independently associated with GDM.

### 3.5. Penalized Regression and Internal Model-Stability Analyses

To evaluate potential small-sample bias and coefficient instability, the primary four-variable model was additionally examined using Firth penalized logistic regression. Penalization resulted in modest attenuation of the effect estimates but did not alter their directions. HOMA-IR remained positively associated with subsequent GDM (OR 1.993, 95% profile-likelihood CI 1.083–3.928; *p* = 0.026), whereas free β-hCG MoM (OR 0.361, 95% CI 0.124–0.917; *p* = 0.031) and IGF-1 (OR 0.871, 95% CI 0.758–0.982; *p* = 0.023) retained inverse associations. The association for TyG remained positive but was borderline after penalization (OR 3.393, 95% CI 0.996–12.508; *p* = 0.051).

In the internal stability analysis, all 2000 stratified bootstrap models converged successfully. The direction of the original regression coefficient was retained in 99.4% of bootstrap samples for HOMA-IR, 97.8% for TyG, 99.2% for free β-hCG MoM, and 99.2% for IGF-1. The corresponding bootstrap median ORs (95% percentile CIs) were 2.209 (1.141–5.160), 4.207 (1.050–22.030), 0.304 (0.080–0.796), and 0.850 (0.708–0.973), respectively. Although the directions of association were highly consistent across bootstrap samples, the wider interval for TyG indicated comparatively greater uncertainty in its coefficient estimate.

### 3.6. Correlation and Multicollinearity Assessment

HOMA-IR and TyG were significantly but moderately correlated in the complete analytic cohort (Pearson r = 0.361, *p* < 0.001; Spearman ρ = 0.372, *p* < 0.001). Multicollinearity diagnostics for the primary four-variable model showed low VIF values and high tolerance values: HOMA-IR, VIF = 1.152 and tolerance = 0.868; TyG, VIF = 1.185 and tolerance = 0.844; free β-hCG MoM, VIF = 1.037 and tolerance = 0.964; and IGF-1, VIF = 1.068 and tolerance = 0.936. These findings indicated no evidence of problematic multicollinearity among the variables included in the primary model.

## 4. Discussion

### 4.1. Principal Findings

Differences between pregnancies with and without subsequent GDM were already apparent in the first trimester and involved several biological domains rather than one isolated pathway. The later-GDM group showed an adverse insulin/lipid–glucose profile together with lower levels of selected placental and IGF-related biomarkers [13,14,16,17,19,21,22,38]. Correlation patterns also varied by group. After adjustment, higher HOMA-IR and TyG were associated with GDM, whereas free β-hCG MoM and IGF-1 showed inverse associations. These findings support the presence of concurrent early alterations in insulin resistance, lipid–glucose metabolism, placental biology, and IGF signaling.

The group-stratified correlation analyses were undertaken to characterize within-group biomarker relationships rather than to demonstrate formal differences between the GDM and control groups. The observed associations may provide preliminary insight into the interplay among placental, metabolic, and IGF-related pathways; however, the subgroup sample sizes, particularly the 30 GDM cases, limit the precision of the correlation estimates. In addition, the number of correlations examined increases the possibility of chance findings due to multiple comparisons. These results should therefore be regarded as exploratory and hypothesis-generating and require confirmation in larger independent cohorts using formal tests of between-group differences in correlation coefficients.

### 4.2. First-Trimester Placental Biomarkers

Women who subsequently developed GDM had lower PAPP-A and free β-hCG concentrations, and free β-hCG MoM retained an independent inverse association in the adjusted analysis. This pattern is compatible with differences in trophoblastic or placental adaptation occurring before diagnostic hyperglycemia [10,13,14,15]. Evidence is strongest for PAPP-A: a recent meta-analysis of more than 90,000 pregnancies found lower first-trimester PAPP-A among women who later developed GDM, although its discriminatory ability as a single marker was modest [13]. Findings for free β-hCG have been less consistent [14]. The present results therefore support an early placental component without suggesting that either analyte should be used alone for diagnosis.

A mechanistic link is plausible because PAPP-A regulates local IGF availability and contributes to biological processes relevant to trophoblast invasion and placental vascular development [11,12]. For free β-hCG, normalization as MoM may better represent gestational-age-adjusted trophoblastic activity than the uncorrected concentration, potentially accounting for the persistence of the MoM association after adjustment. Placental screening markers may therefore be more useful when considered within a broader multivariable context [9,14].

The higher neonatal birth weight observed in the GDM group does not conflict with the lower placental-marker concentrations recorded early in pregnancy. Fetal growth is determined by cumulative influences throughout gestation, and the metabolic milieu associated with GDM later in pregnancy can enhance fetal growth independently of first-trimester placental biomarker levels [39].

### 4.3. The Insulin-like Growth Factor System

Maternal metabolism, placental physiology, and fetal growth are all influenced by the IGF system [11,40,41]. First-trimester IGF-1 was lower in participants who later developed GDM and remained inversely associated with GDM after adjustment. Longitudinal studies have similarly indicated that changes within the IGF axis can occur before clinical GDM becomes evident [38,40]. Given the roles of IGF-1 in glucose homeostasis, insulin-related signaling, and placental function, our finding may represent altered early maternal–placental adaptation rather than a consequence of established hyperglycemia [11,22].

IGFBP-5 showed a different pattern. Its group-level difference did not reach statistical significance, although univariable regression linked higher IGFBP-5 to lower GDM odds. Because only 30 outcome events were available, IGFBP-5 was not carried into the deliberately parsimonious primary model. Direct evidence on circulating IGFBP-5 before GDM diagnosis remains limited, but experimental work implicates this protein in local IGF modulation, placental remodeling, cellular differentiation, and tissue repair [22,41]. Thus, the absence of a strong circulating difference does not rule out local biological effects. Studies integrating serum concentrations with placental expression and downstream signaling would help clarify its role.

### 4.4. Metabolic Biomarkers and Insulin Resistance

The first-trimester metabolic profile also indicated reduced insulin sensitivity and altered glucose–lipid handling before routine GDM testing. Women who later developed GDM had higher fasting insulin, HOMA-IR, triglycerides, TyG, and TG/HDL-C and lower HDL-C, in agreement with previous prospective studies and meta-analytic evidence [16,17,19,21]. Both HOMA-IR and TyG remained associated with GDM in the adjusted model. Although each reflects insulin-resistant metabolism, their inputs differ: HOMA-IR uses fasting glucose and insulin, whereas TyG is derived from glucose and triglycerides [18,36,42,43].

Retention of both HOMA-IR and TyG after adjustment may indicate that they capture overlapping but nonidentical features of early metabolic dysfunction. Previous reports have also identified TyG and TG/HDL-C as readily obtainable indicators of early metabolic risk [19,21]. Together, these findings support further evaluation of a limited set of complementary metabolic markers rather than multiple closely related derivatives.

An important feature of this study is that placental screening biomarkers, conventional metabolic indices, and IGF-axis measurements were evaluated prospectively in the same women. This design permits the different biological domains to be interpreted together rather than in separate cohorts [9,22]. The adjusted relationships of HOMA-IR, TyG, free β-hCG MoM, and IGF-1 suggest potentially complementary information from metabolic, placental, and IGF-related processes. Free β-hCG is already available from routine first-trimester screening, and the metabolic indices are derived from commonly obtained laboratory values, which makes this combination worthy of further study. However, our analyses establish associations and do not constitute validation of a clinical prediction model [10,14,19,43].

The principal associations were broadly preserved after additional adjustment for maternal age, BMI, and parity, suggesting that they were not explained solely by these established maternal risk factors in this cohort. However, given the limited number of GDM events and the number of covariates included, these expanded models should be interpreted cautiously as sensitivity analyses rather than definitive adjusted prediction models.

Firth penalization and bootstrap resampling provided reassurance regarding the directional stability of the primary model, although the TyG estimate remained less precise. These analyses reduce, but do not eliminate, concerns about small-sample bias and overfitting; therefore, the four-variable model should be considered exploratory and hypothesis-generating rather than validated, with confirmation required in larger independent cohorts.

The moderate correlation between HOMA-IR and TyG and the absence of problematic multicollinearity suggest that these indices were not statistically redundant in this dataset. Nevertheless, their conceptual and mathematical overlap warrants cautious interpretation of their individual coefficients, particularly given the limited number of GDM events and the lower precision of the TyG estimate.

### 4.5. Strengths and Limitations

Prospective collection of all biomarker samples before GDM diagnosis is a principal strength because it preserves the temporal relationship between the measured exposures and the later outcome. In addition, placental, metabolic, and IGF-related measurements were obtained from the same participants, allowing direct comparison across biological domains. The adjusted analysis was intentionally structured around a small number of nonredundant markers to examine which relationships persisted when considered jointly.

The findings must also be interpreted in light of several limitations. The study was conducted at one center and the final sample was modest, which reduces precision and limits generalizability. A further important limitation concerns derivation of the final analytic cohort. Although 200 participants completed the 75-g OGTT and GDM status was therefore available for these women, IGF-1 and IGFBP-5 were measured after completion of follow-up only in stored serum samples from the 90 participants with complete clinical follow-up and available specimens. Consequently, complete multidomain biomarker analyses could not be performed in the remaining participants, and their IGF-axis measurements cannot now be recovered because the stored samples are no longer available. Furthermore, retention differed substantially according to subsequent GDM status: 30 of 34 women with GDM and 60 of 166 normoglycemic women were included in the complete-biomarker cohort. This differential attrition introduces a potential risk of selection bias and may limit the representativeness and generalizability of the observed associations. The findings should therefore be considered exploratory and require confirmation in larger prospective cohorts in which biomarker measurements are completed independently of subsequent follow-up availability. Biomarkers were measured at a single first-trimester time point, so longitudinal changes could not be assessed, and placental expression or functional activity of IGF-related proteins was not examined. With only 30 GDM events, the adjusted model was deliberately restricted to four variables; despite this approach, coefficient instability and overfitting remain possible. Finally, model derivation and assessment used the same cohort and no external validation was performed [9,44]. Although sensitivity analyses accounted for maternal age, BMI, and parity, family history of diabetes was not systematically recorded and could not be evaluated as a potential confounder. This residual clinical information gap should be considered when interpreting the adjusted associations.

### 4.6. Future Perspectives and Overall Interpretation

Collectively, these results suggest that pregnancies destined to develop GDM can show measurable biological differences as early as the first trimester. The adjusted analysis retained two metabolic indices (HOMA-IR and TyG), a placental screening marker (free β-hCG MoM), and an IGF-axis measure (IGF-1), supporting continued evaluation of multidomain approaches rather than dependence on a single biomarker. Larger multicenter cohorts should next assess discrimination, calibration, incremental value over established clinical risk factors, and reproducibility in independent populations before any risk-stratification application is considered [9,14,43,44].

## 5. Conclusions

In this prospective cohort, later GDM was associated with a first-trimester profile involving metabolic, placental, and IGF-related measures. In the adjusted analysis, higher HOMA-IR and TyG corresponded to higher GDM odds, whereas higher free β-hCG MoM and IGF-1 corresponded to lower odds.

The results support the concept that early susceptibility to GDM reflects several interacting biological processes rather than a single biomarker. The present cohort cannot establish whether combining these variables improves clinical prediction; this will require formal performance assessment and independent confirmation in larger, multicenter populations.

## Figures and Tables

**Table 1:** Comparison of maternal demographic characteristics, biochemical parameters, first-trimester combined screening markers, and pregnancy outcomes between the control and GDM groups.

Variable	Control Group	GDM Group	*p*
Age (years)	28.6 ± 5.44	29.53 ± 4.92	0.431
Gravida	2 (1–3)	2 (1.75–3)	0.252
Parity	1 (0–1.75)	1 (0.75–1)	0.836
Height (m)	1.61 (1.56–1.67)	1.6 (1.57–1.69)	0.668
Weight (kg)	67.82 ± 12.57	70.73 ± 15.34	0.338
BMI (kg/m^2^)	25.77 ± 4.63	27.08 ± 5.53	0.24
Fasting Glucose (mg/dL)	81.32 ± 7.07	84 ± 8.46	0.116
Insulin (μU/mL)	6.38 (4.32–9.55)	9.6 (6.96–13.33)	0.003 *
HOMA-IR	1.26 (0.79–1.9)	1.95 (1.38–2.57)	0.005 *
HDL-C (mg/dL)	62.5 (50–72.75)	52.5 (42.75–66)	0.025 *
LDL-C (mg/dL)	88.58 ± 23.09	92.03 ± 28.71	0.54
Total Cholesterol (mg/dL)	171.58 ± 27.56	174.83 ± 30.2	0.611
Triglycerides (mg/dL)	100 (83–137)	152 (101.5–198)	0.003 *
VLDL-C (mg/dL)	19.5 (16.25–26.75)	24 (19–35)	0.043 *
TyG index	8.377 ± 0.41	8.68 ± 0.41	0.002 *
TG/HDL-C ratio	1.61 (1.17–2.54)	2.63 (1.92–3.74)	0.002 *
LDL/HDL-C ratio	1.42 (1.05–1.84)	1.67 (1.19–2.18)	0.208
Gestational Age at First-Trimester Screening (weeks)	12.6 (12.3–13.4)	12.6 (12.3–13.2)	0.457
free β-hCG (IU/L)	36.58 ± 16.37	28.85 ± 13.72	0.029 *
free β-hCG MoM	1.11 (0.61–1.61)	0.8 (0.53–1.19)	0.057
PAPP-A (mIU/L)	2.98 ± 1.23	2.16 ± 0.94	0.002 *
PAPP-A MoM	1.08 (0.82–1.36)	0.84 (0.71–1.1)	0.018 *
IGF-1 (ng/mL)	9.96 (7.91–13.55)	8.06 (5.47–11.21)	0.007 *
IGFBP-5 (ng/mL)	134.16 (105.48–197.84)	123.43 (94.05–153.8)	0.092
Gestational Age at Delivery (weeks)	38.85 (38.4–39.4)	38.6 (38.3–39.2)	0.209
Birth Weight (g)	3279.33 ± 350.89	3482.67 ± 362.6	0.012 *

Values are reported as mean ± SD for approximately normally distributed variables or median (IQR) for non-normally distributed variables. Depending on distribution, continuous variables were tested with the independent-samples *t*-test or Mann–Whitney U test. A two-sided *p* < 0.05 was considered statistically significant. Abbreviations: BMI, body mass index; GDM, gestational diabetes mellitus; HDL-C, high-density lipoprotein cholesterol; HOMA-IR, Homeostasis Model Assessment of Insulin Resistance; IGF-1, insulin-like growth factor-1; IGFBP-5, insulin-like growth factor-binding protein-5; LDL-C, low-density lipoprotein cholesterol; MoM, multiples of the median; PAPP-A, pregnancy-associated plasma protein-A; TG, triglycerides; TyG, triglyceride–glucose index; VLDL-C, very-low-density lipoprotein cholesterol. * *p* < 0.05.

## Data Availability

The datasets used and/or analyzed during the current study are available from the corresponding author upon reasonable request.

## Supplementary Materials

The following supporting information can be downloaded at: https://www.mdpi.com/article/10.3390/medicina62091804/s1, Table S1: Correlation analysis of first-trimester combined screening biomarkers with maternal clinical, biochemical, metabolic, and pregnancy outcome variables in the control and GDM groups.
