# Supplementary material for: Integrated First-Trimester Assessment of Placental Biomarkers, Metabolic Indices, and IGF-1/IGFBP-5 in Relation to Subsequent Gestational Diabetes Mellitus: A Prospective Cohort Study
Source: Medicina (Kaunas). 2026 Sep 19;62(9):1804. doi: 10.3390/medicina62091804 (PMC13609306; doi:10.3390/medicina62091804)
Supplement: Supplementary file 1 [file medicina-62-01804-s001.zip › medicina-4547855-supplementary.pdf]

Supplementary Materials

Supplementary Table S1. Correlation analysis of first-trimester combined screening biomarkers with maternal clinical, biochemical, metabolic, and pregnancy outcome variables in the control and GDM groups.

		Control Group						GDM Group					
		free β-hCG	free β-hCG MoM	PAPP-A	PAPP-A MoM	IGF-1	IGFBP-5	free β-hCG	free β-hCG MoM	PAPP-A	PAPP-A MoM	IGF-1	IGFBP-5
free β-Hcg (IU/L)	r	1.000	0.870*	0.211	0.182	0.103	0.113	1.000	0.815*	0.280	0.452*	0.121	0.112
	p		<0.001 S	0.105 P	0.164 P	0.435 S	0.392 S		<0.001 P	0.135 P	0.012 S	0.524 P	0.556 P
free β-hCG MoM	r		1.000	0.222	0.122	0.129	0.133		1.000	0.194	0.323	0.054	0.190
	p			0.088 S	0.352 S	0.326 S	0.312 S			0.305 P	0.082 S	0.779 P	0.314 P
PAPP-A (mIU/L)	r			1.000	0.685*	0.791*	0.587*			1.000	0.607*	0.590*	0.499*
	p				<0.001 P	<0.001 S	<0.001 S				<0.001 S	0.001 P	0.005 P
PAPP-A MoM	r				1.000	0.533*	0.386*				1.000	0.163	0.490*
	p					<0.001 S	0.002 S					0.389 S	0.006 S
IGF-1 (ng/mL)	r					1.000	0.515*					1.000	0.370*
	p						<0.001 S						0.044 P
IGFBP-5 (ng/mL)	r						1.000						1.000
	p												
Age (years)	r	-0.117	0.059	0.009	0.046	-0.165	0.028	0.191	0.158	0.079	0.140	-0.232	-0.115
	p	0.373 P	0.655 S	0.946 P	0.730 P	0.209 S	0.834 S	0.312 P	0.403 P	0.676 P	0.461 S	0.217 P	0.545 P
Gravida	r	-0.156	0.059	-0.124	-0.107	-0.104	-0.037	0.118	-0.021	0.265	0.226	-0.087	-0.057
	p	0.233 S	0.656 S	0.344 S	0.414 S	0.428 S	0.777 S	0.533 S	0.911 S	0.157 S	0.229 S	0.648 S	0.764 S
Parity	r	-0.178	0.029	-0.112	-0.104	-0.113	-0.022	0.140	0.017	0.325	0.246	-0.083	0.133
	p	0.174 S	0.828 S	0.395 S	0.429 S	0.391 S	0.866 S	0.460 S	0.930 S	0.080 S	0.190 S	0.664 S	0.484 S
BMI (kg/m²)	r	-0.110	-0.041	-0.299*	-0.116	-0.174	-0.085	0.081	0.228	-0.264	-0.166	-0.106	-0.230
	p	0.401 P	0.755 S	0.020 P	0.378 P	0.184 S	0.519 S	0.672 P	0.226 P	0.158 P	0.381 S	0.578 P	0.221 P
Fasting Glucose (mg/dL)	r	0.165	0.088	-0.109	0.029	-0.180	-0.007	0.227	0.036	0.163	0.319	-0.067	0.261
	p	0.207 P	0.506 S	0.406 P	0.825 P	0.168 S	0.960 S	0.227 P	0.851 P	0.391 P	0.085 S	0.725 P	0.164 P
Insulin (µU/mL)	r	-0.023	0.064	-0.068	-0.145	-0.085	0.077	0.270	0.336	-0.113	-0.055	0.219	0.173

		Control Group						GDM Group					
		free β-hCG	free β-hCG MoM	PAPP-A	PAPP-A MoM	IGF-1	IGFBP-5	free β-hCG	free β-hCG MoM	PAPP-A	PAPP-A MoM	IGF-1	IGFBP-5
	p	0.863 S	0.628 S	0.606 S	0.268 S	0.521 S	0.559 S	0.148 P	0.070 P	0.552 P	0.772 S	0.245 P	0.359 P
HOMA-IR	r	0.009	0.088	-0.094	-0.169	-0.114	0.077	0.312	0.336	-0.044	<0.001	0.204	0.270
	p	0.946 S	0.506 S	0.476 S	0.197 S	0.387 S	0.559 S	0.093 P	0.070 P	0.816 P	0.999 S	0.280 P	0.148 P
HDL-C (mg/dL)	r	0.048	0.059	0.003	0.063	-0.025	-0.027	-0.183	-0.236	0.085	-0.126	0.450*	0.156
	p	0.714 S	0.652 S	0.983 S	0.634 S	0.849 S	0.838 S	0.332 S	0.209 S	0.657 S	0.508 S	0.013 S	0.411 S
LDL-C (mg/dL)	r	-0.150	0.019	-0.177	-0.093	-0.103	0.150	0.051	0.357	-0.027	0.114	-0.066	0.170
	p	0.254 P	0.887 S	0.177 P	0.479 P	0.432 S	0.253 S	0.787 P	0.053 P	0.887 P	0.549 S	0.727 P	0.370 P
Total Cholesterol (mg/dL)	r	-0.012	0.156	-0.037	0.050	-0.024	0.242	-0.046	0.297	-0.132	-0.078	-0.039	0.112
	p	0.928 P	0.235 S	0.778 P	0.703 P	0.853 S	0.062 S	0.808 P	0.111 P	0.487 P	0.684 S	0.837 P	0.555 P
Triglycerides (mg/dL)	r	-0.171	-0.072	-0.082	-0.133	0.076	0.067	0.171	0.192	-0.233	0.118	-0.336	0.017
	p	0.190 S	0.582 S	0.532 S	0.311 S	0.562 S	0.608 S	0.366 P	0.309 P	0.215 P	0.533 S	0.070 P	0.930 P
TyG index	r	-0.152	-0.063	-0.117	-0.160	0.022	0.039	0.275	0.261	-0.197	0.163	-0.327	0.022
	p	0.246 S	0.635 S	0.373 S	0.221 S	0.866 S	0.766 S	0.141 S	0.164 S	0.296 S	0.389 S	0.078 S	0.909 S
TG/HDL-C ratio	r	-0.104	-0.038	-0.037	-0.119	0.091	0.059	0.177	0.223	-0.222	0.142	-0.459*	-0.112
	p	0.428 S	0.773 S	0.777 S	0.364 S	0.488 S	0.655 S	0.350 S	0.237 S	0.238 S	0.454 S	0.011 S	0.554 S
LDL/HDL-C ratio	r	-0.129	-0.004	-0.082	-0.112	-0.037	0.097	-0.007	0.148	0.016	0.146	-0.265	-0.010
	p	0.326 S	0.978 S	0.531 S	0.395 S	0.777 S	0.460 S	0.971	0.436 S	0.932 S	0.440 S	0.158 S	0.956 S
VLDL-C (mg/dL)	r	-0.113	-0.051	-0.101	-0.084	0.071	0.067	0.180	0.285	-0.244	0.038	-0.201	-0.085
	p	0.390 S	0.698 S	0.442 S	0.522 S	0.591 S	0.610 S	0.341 S	0.126 S	0.194 S	0.844 S	0.286 S	0.654 S
Gestational Age at First-Trimester Screening (weeks)	r	-0.028	0.037	0.537*	0.357*	0.328*	0.237	0.208	0.188	0.184	0.288	-0.147	0.308
	p	0.835 S	0.780 S	<0.001 S	0.005 S	0.011 S	0.068 S	0.271 S	0.319 S	0.331 S	0.123 S	0.439 S	0.098 S
Gestational Age at Delivery (weeks)	r	0.109	0.147	0.263*	0.100	0.243	0.144	0.103	0.036	-0.093	-0.072	-0.136	-0.085
	p	0.405 S	0.262 S	0.043 S	0.447 S	0.061 S	0.271 S	0.588 S	0.851 S	0.627 S	0.703 S	0.474 S	0.654 S
Birth Weight (g)	r	-0.110	-0.182	-0.089	-0.071	-0.085	-0.079	0.284	0.105	-0.140	-0.168	-0.199	0.190
	p	0.403 P	0.163 S	0.498 P	0.592 P	0.519 S	0.548 S	0.128 P	0.580 P	0.462 P	0.376 P	0.291 S	0.314 P

Pearson or Spearman methods were selected according to variable distribution, and the table reports the resulting correlation coefficients (r) with p values. Bold values denote statistical significance at $p < 0.05$. Abbreviations: BMI, body

mass index; GDM, gestational diabetes mellitus; HDL-C, high-density lipoprotein cholesterol; HOMA-IR, Homeostasis Model Assessment of Insulin Resistance; IGF-1, insulin-like growth factor-1; IGFBP-5, insulin-like growth factor-binding protein-5; LDL-C, low-density lipoprotein cholesterol; MoM, multiples of the median; PAPP-A, pregnancy-associated plasma protein-A; TG/HDL-C, triglyceride/high-density lipoprotein cholesterol; TyG, triglyceride–glucose; VLDL-C, very-low-density lipoprotein cholesterol; free β-hCG, free β-human chorionic gonadotropin; LDL/HDL-C, low-density lipoprotein/high-density lipoprotein cholesterol. P, Pearson correlation; S, Spearman rank correlation; r, correlation coefficient; p, p value.
